# How to Study Biofilms after Microbial Colonization of Materials Used in Orthopaedic Implants

**DOI:** 10.3390/ijms17030293

**Published:** 2016-02-26

**Authors:** Lorenzo Drago, Serse Agrappi, Monica Bortolin, Marco Toscano, Carlo Luca Romanò, Elena De Vecchi

**Affiliations:** 1 Laboratory of Clinical Chemistry and Microbiology, IRCCS Galeazzi Orthopaedic Institute, via R. Galeazzi 4, 20161 Milan, Italy; serse.agrappi@gmail.com (S.A.); monica.bortolin85@gmail.com (M.B.); devecchi.elena@gmail.com (E.D.V.); 2 Laboratory of Clinical Microbiology, Department of Biomedical Sciences for Health, University of Milan, via L. Mangiagalli 31, 20133 Milan, Italy; toscano.marco1@gmail.com; 3 Department of Bone and Joint Infections and Reconstructive Surgery, IRCCS Galeazzi Orthopaedic Institute, via R. Galeazzi 4, 20161 Milan, Italy; carlo.romano@grupposandonato.it

**Keywords:** confocal laser scanning microscopy, biofilm, fluorescent stains, images analysis, prosthetic implants

## Abstract

Over the years, various techniques have been proposed for the quantitative evaluation of microbial biofilms. Spectrophotometry after crystal violet staining is a widespread method for biofilm evaluation, but several data indicate that it does not guarantee a good specificity, although it is rather easy to use and cost saving. Confocal laser microscopy is one of the most sensitive and specific tools to study biofilms, and it is largely used for research. However, in some cases, no quantitative measurement of the matrix thickness or of the amount of embedded microorganisms has been performed, due to limitation in availability of dedicated software. For this reason, we have developed a protocol to evaluate the microbial biofilm formed on sandblasted titanium used for orthopaedic implants, that allows measurement of biomass volume and the amount of included cells. Results indicate good reproducibility in terms of measurement of biomass and microbial cells. Moreover, this protocol has proved to be applicable for evaluation of the efficacy of different anti-biofilm treatments used in the orthopaedic setting. Summing up, the protocol here described is a valid and inexpensive method for the study of microbial biofilm on prosthetic implant materials.

## 1. Introduction

In the recent past, it has become evident that knowledge of biofilm composition and architecture is of crucial importance, due to its role in environment, industrial processes and human diseases. Biofilm is defined as a microbial community protected by self-produced polymeric matrix and adherent to various surfaces, such as prosthetic implants used in the clinical setting [1,2]. Biofilm-related infections are known to be more difficult to eradicate in comparison to infections caused by planktonic bacteria [3,4]. Indeed, several studies showed an increase of about 1000 times in resistance to certain antibiotics for bacteria embedded into biofilm matrix, compared to their planktonic counterpart [5]. Over the years, various techniques have been developed in order to highlight and measure the amount of microbial biofilms produced on different substrates. One of the oldest and most widely used method is the quantification of biofilm stained with crystal violet by spectrophotometry [6]. Unfortunately, some reports evidenced the scarce specificity of this semi-quantitative method [7,8], which, with the advances in technology, has been replaced by more complex technologies able not only to sharpen the sensitivity, but also to increase the specificity of biofilm evaluation. Analysis performed by means of confocal laser scanning microscopy (CLSM) is one of the most sensitive and specific assays to evaluate microbial biofilms, able to guarantee a high quality/price ratio. CLSM was initially developed for the study of cell cultures and has been adapted for the study of biofilm since 1995 [9,10,11]. CLSM significantly enhances the spatial resolution of the sample, eliminating the halos due to the light diffused from planes that are out of focus. Moreover, CLSM allows one to focus a laser with high precision on the sample, markedly increasing resolution and depth of the field. One or more lasers, typically semiconductors, constitute its light source for each excitation frequency requested. A computer system handles the mechanism directing the light beam. The images are obtained by synchronizing the excitation beam and the detection device. The different fluorescent markers can be depicted with different colors, allowing one to appreciate the three-dimensionality of the sample. Nonetheless, differences between the layer of cells and the visible structure of the exopolysaccharides matrix (EPS), which represents the major component of biofilm, notably complicate analysis of biofilm. For this reason, preliminary studies were initially conducted only to display biofilm without performing any quantitative measurement in relation to the thickness of the matrix and the number of embedded bacteria. Other studies using genetically engineered bacteria to quantify biofilm, raised some concerns about the use of strains artificially created in laboratory, which might have no relation with clinical isolates [12,13]. In recent years, research on the use of CLSM for the analysis of microbial biofilms has taken many steps forward, developing new dyes and dedicated graphic software able to quantify the biofilm [14,15]. Commercial kits are now available on the market, favoring the repeatability of analysis by making sample treatment uniform. The major limitation in the study of biofilms grown on prosthetic implants is the lack of standardized *in vitro* models and of easy to use assays. For this reason, the need to evolve and adapt these techniques to specific purposes, in the attempt to develop a method easy to perform, even using different prosthetic implants as growth support, is quite urgent.

The aim of the present study was to develop a CLSM technique that not only allows one to highlight biofilms, but also measures biomass volume and quantifies living and dead microorganisms embedded in a biofilm grown on sandblasted titanium.

## 2. Results

The protocol was validated by the analysis of several images acquired from biofilms formed on sandblasted titanium discs by *Staphylococcus aureus*, *Pseudomonas aeruginosa* and *Candida albicans*. Biofilms were evaluated by using the FilmTracer™ LIVE/DEAD^®^ Biofilm Viability Kit (Invitrogen Ltd., Paisley, UK), which consists of a mixture of the green-fluorescent nucleic acid stain SYTO^®^ 9 and the red-fluorescent nucleic acid stain propidium iodide (PI). These stains differ in both their spectral characteristics and ability to penetrate healthy microbial cells. The SYTO^®^ 9 stain generally labels all microorganisms in a population, either those with intact membranes and those with damaged membranes. In contrast, PI penetrates only microorganisms with damaged membranes, reducing the SYTO^®^ 9 fluorescence when both dyes are present. Thus, with an appropriate mixture of the SYTO^®^ 9 and PI stains, microorganisms with intact cell membranes (*i.e.*, live) are stained in fluorescent green, whereas microorganisms with damaged membranes (*i.e.*, dead) are stained in fluorescent red.

**Figure 1:**
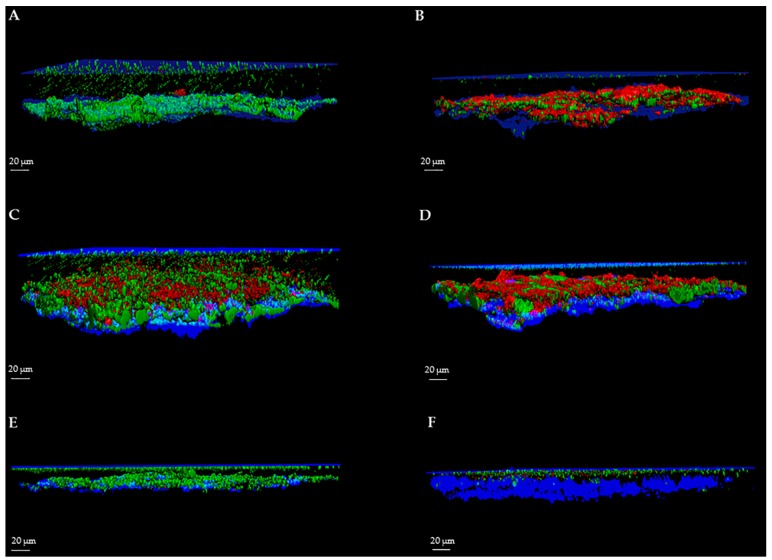
3D image of *S. aureus*, *P. aeruginosa* and *C. albicans* biofilms using SYTO^®^ 9 and propidium iodide (PI). 3D reconstruction of *S. aureus*, *P. aeruginosa* and *C. albicans* untreated and treated biofilms by CLSM. Biofilms were grown for 72 h and then stained with SYTO^®^ 9 and PI. (**A**) *S. aureus* biofilm formed on sandblasted titanium disc; (**B**) *S. aureus* biofilm treated with an anti-biofilm substance; (**C**) *P. aeruginosa* biofilm formed on sandblasted titanium disc; (**D**) *P. aeruginosa* biofilm treated with an anti-biofilm substance; (**E**) *C. albicans* biofilm formed on sandblasted titanium disc; (**F**) *C. albicans* biofilm treated with an anti-biofilm substance.

**Figure 2:**
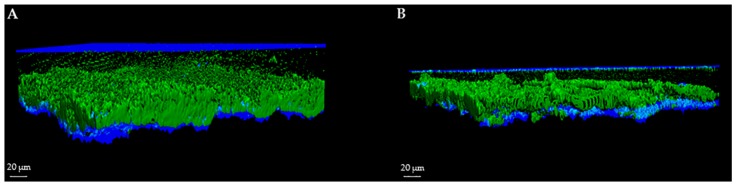
3D image of *P. aeruginosa* biofilm using SYTO^®^ 9 alone. 3D reconstruction of *P. aeruginosa* untreated and treated biofilm by confocal laser scanning microscopy (CLSM). Biofilm were grown for 72 h and then stained with SYTO^®^ 9. (**A**) *P. aeruginosa* biofilm formed on sandblasted titanium disc; (**B**) *P. aeruginosa* biofilm treated with an anti-biofilm substance.

For each image, a specific user-friendly software (Imaris, Bitplane AG, Zurich, Switzerland) allowed the reconstruction of a 3D image of the portion of the disc. Volume rendering was obtained by initially selecting only the blue color channel corresponding to titanium and coverslip surfaces, and configuring them as the lower and the upper sides of the image. Then, the green and red channels, corresponding respectively to the cell volume of living and dead microorganisms within the biofilm, were added to the rendering. Finally, the intensity of the blue channel corresponding to the titanium and coverslip surfaces was adjusted to decrease overlapping with the other channels and to better highlight the volume of live and dead cells. As shown in Figure 1 and Figure 2, the elaboration of the acquired images provide visualization of the disc’s surface, biofilm matrix and biofilm-embedded microorganisms.

To better evaluate the applicability of our method for the assessment of the activity of anti-biofilm treatments, we determined the efficacy of three different compounds in reducing biofilm, one for each microbial strain. All the analyses of the biofilms were easily reproducible.

### 2.1. Quantification of Total Biomass Volume

Quantification of total biomass volume was performed by calculating the mean value of biofilm thickness multiplied for the total surface of the acquired portions through the software Leica LAS AF (Leica Microsystems CMS GmbH, Mannheim, Germany). Table 1 reports the measurements of *S. aureus*, *P. aeruginosa* and *C. albicans* biomass volumes conducted on different control and treated discs. Results are expressed as mean ± SD of data obtained from three different titanium discs. For each disc, at least four microscopic fields were analyzed and means calculated.

As shown, measurement of biofilm volume was quite reproducible for all microorganisms. As reported in Table 1, also the determination of biomass after anti-biofilm treatments, which caused a sustained reduction of microbial mass, produced repeatable results with CV% ranging from 1.89% to 4.81% (mean CV%: 3.41%). Treatments caused a significant reduction of Biomass volume (BV) in respect to control samples (*p* < 0.001).

### 2.2. Quantification of Volume Occupied by Microbial Cells into Biofilm

**Figure 3:**
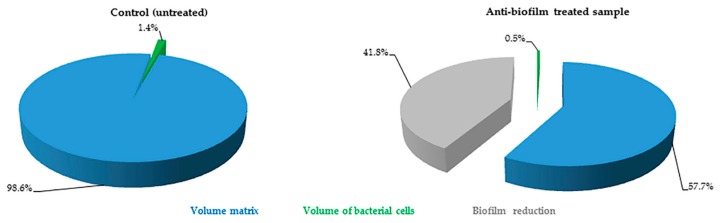
*P. aeruginosa* biofilm composition of untreated (control) and treated sample obtained using SYTO 9^®^ alone. Pie charts represent the *P. aeruginosa* biomass components proportion obtained from CLSM images analysis performed by Fiji software (Fiji, ImageJ, Wayne Rasband National Institutes of Health, Bethesda, MD, USA). Blue: biofilm matrix; green: total cells within the biofilm. For the treated sample, the loss of biofilm caused by treatment with an anti-biofilm substance in comparison with control is reported in gray.

**Figure 4:**
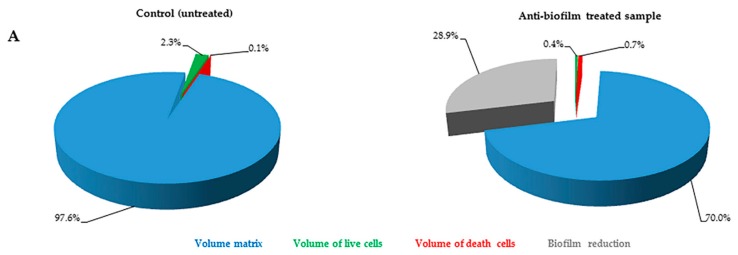

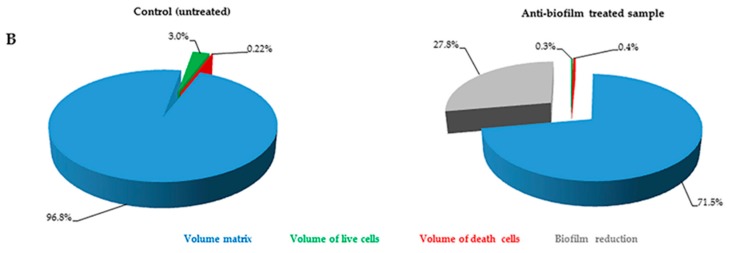
*S. aureus* (**A**) and *C. albicans* (**B**) composition (%) of untreated (control) and treated samples obtained using SYTO^®^ 9 and PI. Pie charts represent the biomass components proportion obtained from CLSM images analysis performed by Fiji software. Blue: biofilm matrix; green: live cells within the biofilm; red: dead cells within the biofilm. For treated samples, loss of biofilm caused by an anti-biofilm substance in comparison with control is reported in gray.

The FilmTracer™ LIVE/DEAD^®^ staining properties, together with the instrumental set-up allowed quantification of the volume occupied by all of the cells embedded in the matrix. In particular, using SYTO^®^ 9 stain alone, it was possible to stain all microbial cells (live and dead) and consequently to quantify the volume occupied by all the cells into the matrix (Figure 3). Furthermore, using SYTO^®^ 9 and PI together, the ratio between live and dead cells was calculated, thus simultaneously evaluating anti-biofilm and antimicrobial activity of any compound (Figure 4).

## 3. Discussion

A great challenge of the 21st century is the development of novel and effective strategies for the treatment of infections sustained by microbial biofilms.

In this paper, we presented a protocol for *in vitro* evaluation of biofilm grown on sandblasted titanium used for prosthetic implants. This method has been developed to respond to the need for a highly specific and sensitive procedure for investigating the activity of anti-biofilm agents at a low cost, and to evaluate biofilms in diagnosis of biofilm-related infections. Since the ability to growth in biofilm may differ depending on the substrate, it is necessary to implement classical methods for the study of biofilms, introducing those materials that are commonly used in prosthetic orthopaedic surgery, and to use microbial strains clinically isolated from prosthetic infections without introducing any genetic manipulation.

For many years, the anti-biofilm activity was investigated by semi-quantitative methods. In 1980s, Christensen *et al.* [6] developed the first semi-quantitative method for biofilm evaluation, measuring the absorbance of crystal violet dye included into the biofilm. This method is still widely used [16,17,18], thanks to the ease of the procedure and the low costs, although, by time, experience pointed out its low sensitivity and specificity, due to the fact that crystal violet is a basic dye that readily binds to negatively charged molecules (and thus to acidic polysaccharides in the extracellular matrix) and the wide variability of results due to the variable extraction of the dye by ethanol. [19]. Moreover, this assay is performed on air-dried biofilm and it is unable to yield any information on biofilm three-dimensional architecture. On the contrary, with the aid of CLSM, biofilms can be studied in their natural hydrated state, with no requirement for desiccation nor other invasive and destructive processing methods such as chemical fixation or embedding techniques.

Indirect methods are simple and fast for biofilm analysis, and one of them, the count of microbial cells embedded in biofilm, is one of the most commonly used technique [20]. However, this kind of analysis presents some limitations and lack of precision, as microorganisms are detached with physical or chemical treatments that may destroy the biomass, thus altering vital microbial counts. Sonication, for instance, is widely used to detach and recover bacteria embedded in biofilm, but it is known that this treatment is not very effective towards *Staphylococcus epidermidis* biofilm [21].

For these reasons, with improvements in technology, semi quantitative methods have been replaced by more complex procedures, using instrumentation able to increase sensitivity and specificity. Initially, the use of scanning electron microscopy (SEM) seemed to solve many of the problems presented by the older methods, in that it provided excellent images of biofilm, with high resolution power and magnification [22,23]. However, the lack of ability to perform quantification, the high cost of instrumentation, the long time needed for sample preparation and the need for skilled personnel have greatly restricted its use.

CLSM analysis is undoubtedly one of the most sensitive and specific techniques for visualization and analysis of biofilm structure. CLSM is characterized by high sensitivity and specificity and allows 3D digital recreation of image of whole portions of biofilm with high resolution. However, some problems in this CLSM application have been identified, especially due to the particular structure of the biofilm that makes its analysis unwieldy and difficult to perform.

Our method has the purpose of facilitating these analyses, still providing good results with simple procedures. In the last two decades, graphics dedicated software or particular biofilm treatments have also been developed to deeply analyze biofilms [14,15,24].

In order to facilitate the graphics processing of acquired sections, we opted for the use of a user-friendly software (Imaris, Bitplane AG).

From the technical point of view and in terms of processing algorithms, Fiji software (Fiji, ImageJ, Wayne Rasband National Institutes of Health) does not present substantial differences if compared to other software developed for the study of biofilm (*i.e.*, COMSTAT) and, as previously mentioned, its use is simple and intuitive. Moreover, it is a freely available software, thus not requiring license costs.

Recently, Khajotia *et al.* [9] have developed an interesting approach to biofilm evaluation, allowing quantification of the three main components of *Streptococcus mutans* biofilm: EPS, nucleic acids (cells) and proteins.

Differently from the method described by Khajotia *et al.* [9], the use of a high acquisition frequency (700 Hz) in simultaneous acquisition mode allowed us to avoid false co-localization of fluorescence due to microbial movement. Moreover, the use of a third channel used in reflection mode, allows the distinction of the support from the biofilms mass, and enable observation of the interior of the materials ridges (such as sandblasted titanium). To our knowledge, this is the first method that allows the simultaneous visualization of biofilms and of the substrate used as support for biofilm formation (e.g., titanium). In fact, usually, micrographs represent two- or three-dimensional projections of biofilms without displaying the underlying supporting materials. This is crucial for prosthetic joint and other device-related infections, where the study of interactions between implant surface and bacteria is of outstanding importance.

The fluorescent staining used for this protocol was the FilmTracer™ LIVE/DEAD^®^ Biofilm Viability kit, a simple and reliable staining for both gram-positive and negative bacteria, and fungi like *C. albicans*, which has been successfully used to stain biofilm by several authors [25,26,27,28,29].

Results obtained in this study show that our technique can be used for a thorough analysis of the microbial biofilms developed on prosthetic implants. Here we have presented two modalities to quantify the volume occupied by microbial cells embedded in the biofilm. Quantification of all cells (both live and dead) represents an easy way to analyze biofilm. However, the use of the two fluorescent dyes provides a complete and thorough analysis of biofilm, although, in some cases, differences in biofilm biochemical composition may hamper determination of the amount of PI bound to dead cells. Regardless, even when only total cell volumes were measured, a decrease in cell volume in samples treated with anti-biofilm substances was observed.

Since biofilms have an irregular structure made of canyons and peaks, we recommend obtaining at least four acquisition for each sample to ensure highly precise and reliable data. Thanks to its ease of use and its good adaptability, this protocol can be routinely applied, for example, to evaluate the effect of substances on biofilm grown on different prosthetic implants used in clinical practice.

## 4. Materials and Methods

### 4.1. Microbial Strains

The protocol was set up by using one strain of methicillin-resistant *S. aureus* and one strain of *P. aeruginosa*, isolated from two patients undergoing septic revision surgery of the knee and hip prostheses, respectively, and one strain of *C. albicans*, isolated from a patient with wound drainage after hip replacement. Surgery was performed at the Center for Reconstructive Surgery of Bone and Osteoarticular Infections of Galeazzi Orthopaedic Institute (Milan, Italy). Microorganisms were identified at Laboratory of Clinical Chemistry and Microbiology of the same Institute. Microbial identification was performed by means of biochemical assays (Vitek 2; Biomerieux, Marcy l’Etoile, France) and, only for bacterial strains, further confirmed by DNA sequencing of about 80 bp of variable regions V1 and V3 of the 16S rRNA gene by Pyrosequencing (PSQ96RA, Diatech, Jesi, Italy), as previously described [15,16].

### 4.2. Screening for Biofilm Production

Screening for biofilm production was conducted on 96-wells polystyrene microtiter plates according to Christensen *et al.* [6]. For each sample, 20 µL of an overnight microbial suspension were added to 180 µL of Tryptic Soy Broth (TSB) in at least 3 wells of the microtiter plate. Overnight incubation was carried out aerobically at 37 °C, then medium was refreshed in order to remove not adherent microorganisms, and plates were further incubated for 72 h at 37 °C. Negative controls, comprising wells containing 200 µL of TSB without microbial suspension, were also prepared under the same conditions. At the end of the incubation period, medium was removed, and three washes with Phosphate Buffered Saline (PBS) were performed in order to remove microorganisms not included into biofilm. After air-drying, each well was stained with 200 µL of 3% crystal violet solution for 10 min, then the excess of dye was removed with three washes with PBS. Once dried, 200 µL of absolute ethanol were added to each well, in order to solubilize the dye attached to biofilm. The amount of biofilm produced was determined by spectrophotometric reading at a wavelength of 595 nm using a microplate reader (Multiskan FC, Thermo Scientific; Milan, Italy). Strains were classified as strong producers of biofilm by comparing their optical density with that of the negative control [6].

### 4.3. Biofilm Formation on Prosthetic Material

Sandblasted titanium discs (20 mm diameter × 6 mm thickness; Adler Ortho BATCH J04051) were used as substrates for biofilm formation. Discs were placed into a 6-well plate, with each well containing 4.8 mL of TSB and inoculated with 200 µL of a 0.5 McFarland turbidity microbial suspension. Plates were incubated at 37 °C for 72 h on an orbital shaker, in order to obtain a visible biofilm mass. Before staining, not adherent microorganisms were removed by three washings with sterile saline.

### 4.4. Fluorescent Staining

Staining with FilmTracer™ LIVE/DEAD^®^ Biofilm viability kit (Molecular Probes, Life Technologies Ltd.) was performed according to the instructions provided by the manufacturer. Briefly, a working solution of fluorescent stains was prepared by adding 3 μL of SYTO^®^ 9 stain and 3 μL of PI stain to 1 mL of filter-sterilized water. Two hundreds μL of staining solution were deposited on disc surface and, after 15 min incubation at room temperature in the dark, samples were washed with sterile saline for removing the excess dyes and rinsed with water from the base of the support material.

### 4.5. CLSM Analysis

Stained biofilms were examined with a confocal laser microscope (Leica model TCS SP5; Leica Microsystems CMS GmbH, Mannheim, Germany) using a 20× dry objective (HC PL FLUOTAR 20.0 × 0.50 DRY) plus a 2× electronic zoom. In order to minimize the air contact and maintain constant sample moisture condition, a coverslip was used on the specimen.

A 488 nm laser line was used to excite SYTO^®^ 9, while the fluorescent emission was detected from 500 to 540 nm. PI was excited with 561 nm laser line and its fluorescent emission was detected from 600 to 695 nm. In order to avoid false co-localization of fluorescence due to microbial movement, we opted for a simultaneous acquisition mode of the two channels, in which the laser beam scanned the visual field at a frequency of 700 Hz. Moreover, using a third laser line (633 nm) in reflection mode, it was possible to determine with high accuracy both titanium disc (starting acquisition point) and coverslip (ending acquisition point) reflecting surfaces. Images from at least four randomly selected areas were acquired for each disk. For each of them, sequential optical sections of 2 µm were collected along the *z* axis over the complete thickness of the sample to be subsequently analyzed, quantified by Fiji software (Fiji, ImageJ, Wayne Rasband National Institutes of Health) and rendered into 3D mode by Imaris software (Imaris, Bitplane AG).

### 4.6. CLSM Quantification

The images acquired during the experimental session were processed through a segmentation algorithm (Fiji, ImageJ, Wayne Rasband National Institutes of Health) capable of separating the signals between the background and the sample, in order to obtain a proportionality between the number of microorganisms and the fluorescent signal. More precisely, the algorithm measured the volume occupied by the fluorescent signal recorded in each pixel with value exceeding a threshold on the scale of gray tones that was established to distinguish a specific signal from the background noise. Above this classification, a dimension filter was subsequently applied, using a variable size depending on the type of microorganism used. This filter size represented the approximate value of the volume of the microbial species used for measurements. A variation (%) on the threshold value calculated by Fiji software was applied to better reflect the distinction of microorganisms from background. The software calculated the volume occupied by live microorganisms, by measuring the volume occupied by the relevant fluorescence. The measurement of the volume of the scanned sample was performed by manual calculation of the mean value of biofilm thickness multiplied for total surface of portions acquired through the software Leica LAS AF.

### 4.7. Statistical Analysis

Results of biomass volume obtained by CLSM images analysis are presented as mean ± SD. Repeatability of data is expressed as coefficient of variation (CV%). A *p*-value equal or less than 0.05 was considered as statistically significant.

## 5. Conclusions

Summing up, the protocol here described is a valid and inexpensive method for the study of microbial biofilm on prosthetic implant materials.

## Figures and Tables

**Table 1:** Mean and standard deviation (SD) values of Biomass Volume (BV), Coefficient of Variation (%) of controls (untreated samples) and samples treated with anti-biofilm substance.

Microorganism	BV (µm^3^) ± SD (Controls)	CV% (Controls)	BV (µm^3^) ± SD (Anti-Biofilm Treatment)	CV% (Anti-Biofilm Treatment)	*p*-Value (Treated *vs.* Controls)
*S. aureus*	6.52 × 10^6^ ± 1.81 × 10^5^	2.78%	4.64 × 10^6^ ± 2.23 × 10^5^	4.81%	0.00001
*P. aeruginosa*	1.54 × 10^7^ ± 5.19 × 10^5^	3.37%	8.98 × 10^6^ ± 3.55 × 10^5^	3.95%	0.000001
*C. albicans*	5.23 × 10^6^ ± 9.88 × 10^4^	1.89%	3.74 × 10^6^ ± 1.37 × 10^5^	3.66%	0.000005
